# Real-time ventilation feedback devices for out-of-hospital cardiac arrest: a review of the literature

**DOI:** 10.29045/14784726.2025.9.10.2.24

**Published:** 2025-09-01

**Authors:** Cameron Barcroft, Andrew Crow, Caitlin Wilson

**Affiliations:** Yorkshire Ambulance Service NHS Trust ORCID iD: https://orcid.org/0009-0005-7565-3078; University of Bradford; Yorkshire Ambulance Service NHS Trust; University of Hertfordshire ORCID iD: https://orcid.org/0000-0002-9854-4289

**Keywords:** artificial respiration, out-of-hospital cardiac arrest, sensory feedback

## Abstract

**Introduction:**

In the United Kingdom, ambulance services attempt resuscitation on 30,000 people per year, with fewer than 9% surviving and leaving hospital. Correct ventilation during out-of-hospital cardiac arrest (OHCA) is essential, as both hypo- and hyperventilation are linked to increased mortality. Despite this, ventilations are frequently given outside of recommended guidelines. Devices providing real-time feedback on ventilations aim to improve performance. While systematic reviews show that real-time feedback devices improve chest compression performance, evidence regarding ventilation feedback devices (VFDs) has not yet been synthesised. This literature review aimed to synthesise evidence on the effects of VFDs in OHCAs.

**Methods:**

Databases searched in March 2025 included MEDLINE, CINAHL and Embase. Inclusion criteria were papers published after 1 January 2018, in English, involving adults, focused on clinical practice or simulated OHCA and employing primary research with interventional study designs. The intervention criteria required a VFD that measured and provided feedback on both tidal volume and ventilation rate. Study quality was assessed using the Critical Appraisal Skills Programme checklist. Methods for synthesis included a narrative summary of findings.

**Results:**

The searches yielded 793 results. Nine studies met the inclusion criteria: seven simulation studies and two real-world studies. Simulation studies confirmed that ambulance clinicians often did not meet advanced life support guidelines for ventilations. Introducing VFDs significantly improved compliance, accuracy and precision of delivered ventilations in simulated OHCA scenarios. Real-world studies found an increase in ventilation compliance; however, the study examining patient outcomes was of low quality and did not find a statistically significant effect.

**Conclusion:**

The evidence suggests that VFDs are beneficial in simulated OHCA. Real-world studies suggest that the increase in ventilation performance may not be as significant as shown in simulation studies, and their effect on clinical outcomes has not yet been adequately explored.

## Introduction

In the United Kingdom (UK), the annual incidence of out-of-hospital cardiac arrest (OHCA) is approximately 55 per 100,000 people, with National Health Service (NHS) ambulance services attempting resuscitation on approximately 30,000 people per year. Survival rates, however, remain low, with only 9% surviving to hospital discharge (Perkins et al., 2021).

The Resuscitation Council UK sets guidelines and quality standards for cardiopulmonary resuscitation (CPR) in the UK. The latest adult guidelines state that an endotracheal tube or a supraglottic airway device should be inserted and patients ventilated at 10 breaths per minute, with each ventilation delivered over one second to achieve an approximate tidal volume of 500–600 ml (Soar et al., 2021, 2024).

Correct ventilation is essential, as it has been suggested that both hypoventilation and hyperventilation are associated with increased mortality. Hypoventilation causes inadequate gas exchange within the lungs, leading to hypoxaemia, hypercapnia and acidosis (Cordioli et al., 2016). Hyperventilation has been linked to increased intrathoracic pressures, which impair haemodynamics and reduce cerebral artery blood flow, worsening neurological outcomes (Steiner et al., 2004). Although there are currently no studies exploring the impact of hyperventilation on patient outcomes (Kirk et al., 2023), animal studies suggest it may negatively impact survival rates and neurological outcomes (Aufderheide et al., 2004).

Despite the possibility of harm, ventilations are frequently given outside of recommended guidelines, with hyperventilation consistently occurring in OHCA (Kirk et al., 2023). In their observational study, Park et al. (2013) suggested that this may be due to a multitude of human factors, such as lack of experience, tiredness or lack of leadership. Similar reasons have been linked to the inadequate delivery of CPR, where the introduction of real-time feedback devices has led to greater adherence to guidelines (Gugelmin-Almeida et al., 2021; Lyngby et al., 2021). It is now possible to measure ventilations using a real-time ventilation feedback device, highlighting the potential opportunity to improve clinical practice. The aim of this literature review was to synthesise published evidence on the effects of VFDs and explore if the introduction of this technology could be beneficial to clinical practice.

## Methods

### Search strategy

This literature review started with a scoping search using the NHS Knowledge and Library Hub search engine and AMBER, the ambulance research repository, performed by the primary researcher (CB), using the keywords ‘ventilation’ and ‘feedback’. This identified several journal articles on VFDs and highlighted keywords for the formal literature search. A search for systematic reviews was undertaken using the Cochrane Library, with none identified.

MEDLINE, CINAHL and Embase databases were searched on 2 March 2025. We used a modified population intervention comparison outcome (PICO) tool to define search terms. Including either a ‘Comparison’ or an ‘Outcome’ term led to a reduction in the number of articles returned and excluded several relevant papers identified in the scoping search. Therefore, the final search strategy followed a population intervention strategy. The databases were title and abstract searched using the search terms shown in Table 1 combined with Boolean operators.

**Table 1.** The population and intervention terms utilised in database searching.

Population		Intervention
Paramed*	Emergency Medical Systems	Ventilation feedback
Pre-hospital	EMT	Ventilation device
Prehospital	EMTs	Feedback device
MH “Out-of-Hospital Cardiac Arrest”	MH “Emergency Medical Technicians”	Ventilation monitoring
Out of Hospital cardiac arrest	Emergency Medical Technician	Ventilation monitor
Out of Hospital	MH “Emergency Responders”	Amflow
Out-of-Hospital	Emergency Responder	VQI
OOH	CPR	Ventilation quality indicator
OOHCA	MH “Cardiopulmonary resuscitation”	Ventilation feedback device
Cardiac Arrest	MH “Resuscitation”	Ventilation feedback devices
MH “Heart Arrest”	OHCA	VFD
EMS	ALS	Accuvent
EMS System	Advanced Life Support	EOLife
EMS Systems	BLS	“Real BVM”
MH “Emergency Medical Services”	Basic Life Support	
Emergency Medical Service	MH Advanced Cardiac Life Support	
Emergency Medical System	Respiratory arrest	

Appropriate Embase subject headings replaced keyword searches where possible, and MeSH terms were substituted for relevant subject headings or keywords. Multiple synonyms and brand names for VFDs were utilised, as a consensus name for this technology has not been reached.

### Eligibility criteria and selection process

CB screened papers per the criteria outline in Table 2.

**Table 2.** Inclusion and exclusion criteria, with justifications.

Inclusion	Exclusion	Justification
Papers published after 1 January 2018	Papers published before 1 January 2018	This date cutoff point ensured the inclusion of the most contemporaneous research in this evolving field. It also streamlined the screening process, making it feasible for a single researcher to manage.
English language	Studies not available in English language	Funding was not available to translate studies published in a foreign language and therefore could not be included.
Paper includes a ventilation feedback device that measures and provides feedback on both tidal volume and ventilation rate	Paper does not include a ventilation feedback device that measures and provides feedback on both tidal volume and ventilation rate	Current guidelines define ventilation criteria by both ventilation rate and tidal volume (Soar et al., 2021). The most applicable devices to practice should therefore provide feedback on both aspects.
Feedback device used in the context of a real or simulated cardiac or respiratory arrest	Feedback device not used in the context of a real or simulated cardiac or respiratory arrest	The primary intended use case for VFDs is in OHCA.
Adult population	Paediatric or neonatal population	Adult cardiac arrests make up the majority of resuscitations performed by ambulance services. As such, limiting the literature review to an adult population ensured that the results were applicable to the largest demographic and could be compared.
Primary research	Non-primary research, abstracts, editorials, addendums, research proposals and grey literature	An a priori decision was made not to include grey literature, abstracts or research proposals, as they have not been peer reviewed and therefore are low-quality evidence.
Interventional study designs including RCTs, simulation studies, cohort studies and case-control studies	Observational and descriptive study designs	Observational and descriptive study designs were excluded from the review to maintain a focus on interventional evidence, ensuring robust conclusions about the intervention’s efficacy and impact.
Human or human simulation studies	Animal studies	The results of animal studies cannot be directly applied to a human population group.

Articles were abstract screened and duplicates were manually removed. Included articles were reference list and citation searched, yielding one further article that was not indexed in the above databases.

A PRISMA flowchart was then generated using the online tool provided by Haddaway et al. (2022).

### Data extraction process and data items

CB extracted data from the included studies. This included demographic data (i.e. study country, number of participants), participant details (i.e. participant skill set, training undertaken), intervention design (i.e. ventilation feedback device used, intervention setting, asynchronous versus continuous ventilations) and study outcomes (i.e. ventilations in target for tidal volume and rate).

Critical analysis was performed by CB on each paper using the relevant Critical Appraisal Skills Programme (2021) checklist.

### Synthesis method and effect measures

Studies were synthesised using narrative synthesis (Rodgers et al., 2009), which involved systematically collecting and thematically organising findings from relevant studies, summarising results descriptively and comparing key points. The Synthesis Without Meta-analysis (SWiM) reporting guidelines were followed (Campbell et al., 2020), and studies were grouped by setting (simulation or clinical practice) to reflect differences in context and implementation. Risk ratios were selected as the standardised metric for outcomes to enable consistent comparison across studies. Intervention effects were transformed as required, using Cochrane Handbook guidance (Higgins et al., 2023) to maintain comparability. Given study heterogeneity and incomplete variance reporting (McKenzie & Brennan, 2019), we summarised effect estimates descriptively and did not perform a meta-analysis. High-quality studies were prioritised for synthesis to enhance reliability.

Scott et al. (2021) were approached to provide missing data; however, they were unable to provide this as they do not have approvals in place to reanalyse or share raw data. Lee et al. (2023) were approached to clarify inconsistencies in the reporting of their study results; however, no response was received.

## Results

**Figure 1:**
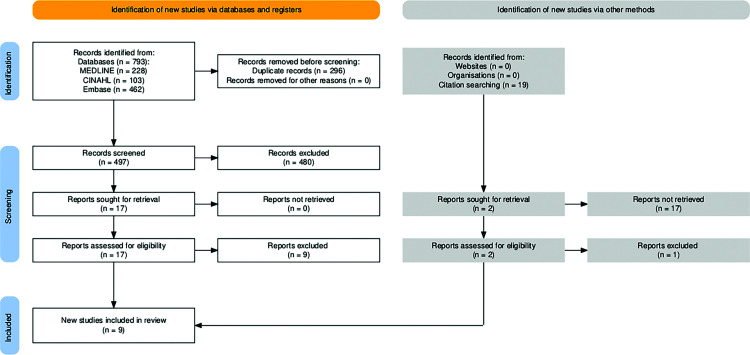
Figure 1. PRISMA flowchart.

The search yielded 793 results. After removing duplicates and performing title, abstract and full-text screening, nine articles were included in the final review. A PRISMA flowchart (Figure 1) was generated (Page et al., 2021).

### Study characteristics

Nine studies were included in this review, comprising seven simulation studies and two clinical trials. Studies were from the United States (Gould et al., 2020; Khoury et al., 2019; Scott et al., 2021), South Korea (Heo et al., 2020; Kim et al., 2020; Lee et al., 2023), Denmark (Lyngby et al., 2021), Canada (Drennan et al., 2024) and the UK (Charlton et al., 2021).

The studies varied in design, including three randomised crossover studies (Heo et al., 2020; Khoury et al., 2019; Kim et al., 2020), four before-and-after studies (Charlton et al., 2021; Drennan et al., 2024; Gould et al., 2020; Scott et al., 2021) and two randomised controlled trials (Lee et al., 2023; Lyngby et al., 2021). Sample sizes ranged from 13 (Heo et al., 2020) to 221 (Drennan et al., 2024), with participants including paramedics, physicians and ambulance staff. Most studies used the ZOLL AccuVent (Charlton et al., 2021; Drennan et al., 2024; Gould et al., 2020; Lee et al., 2023; Lyngby et al., 2021; Scott et al., 2021), while others evaluated Amflow (Kim et al., 2020) or custom devices (Heo et al., 2020; Khoury et al., 2019).

Seven of the studies were partially funded by medical device manufacturers, ranging from the supply of devices (Charlton et al., 2021) to responsibility for data collection and write-up (Gould et al., 2020; Lyngby et al., 2021), or co-authors with pending patent applications (Scott et al., 2020).

These conflicts of interest were appropriately disclosed; however, this does necessitate cautious interpretation.

### Results of individual studies and synthesis

**Figure 2:**
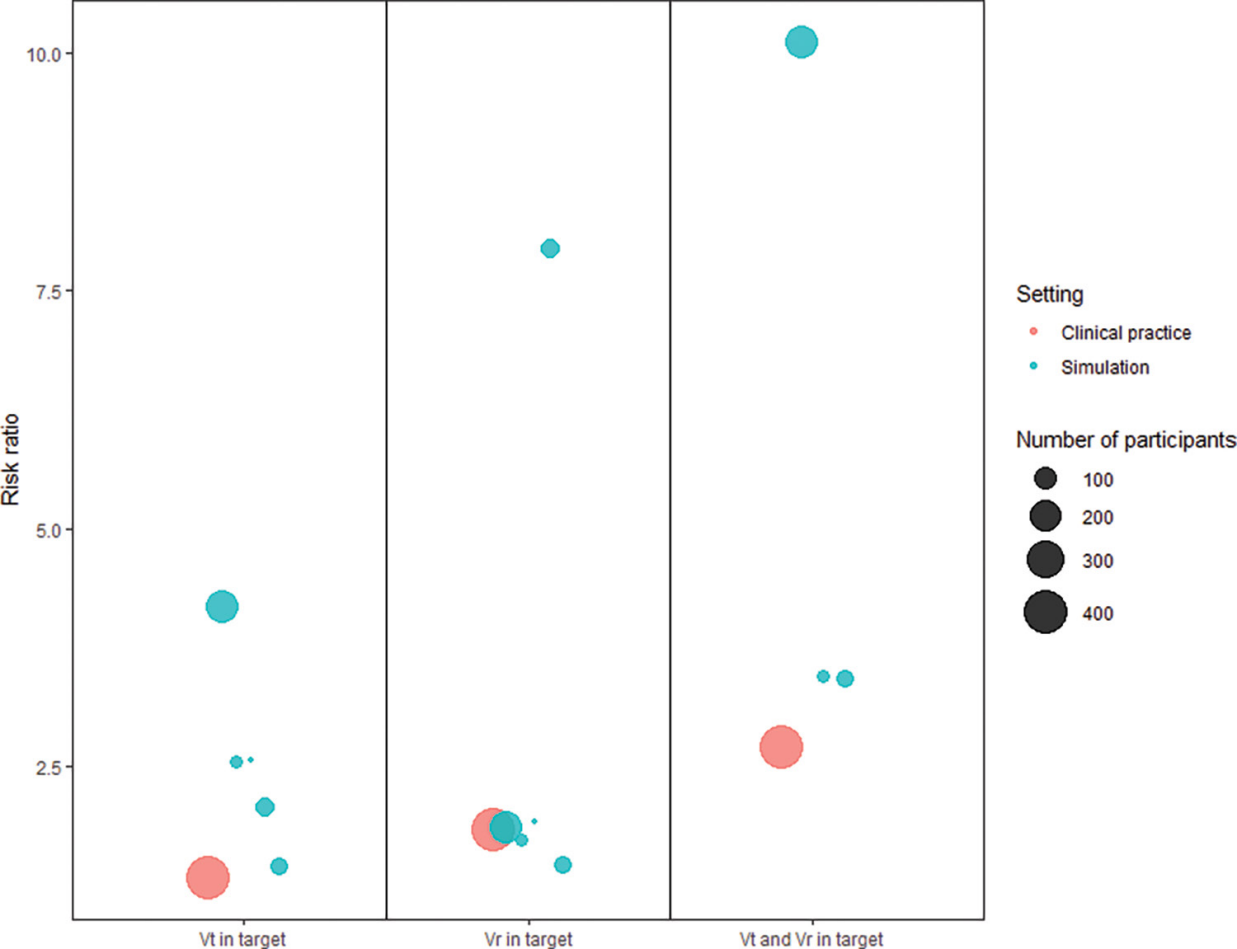
Figure 2. Bubble plot of individual study results.

Six studies (Charlton et al., 2021; Drennan et al., 2024; Gould et al., 2020; Heo et al., 2020; Kim et al., 2020; Lyngby et al., 2021) focused on compliance with ventilation guidelines, assessing the proportion of ventilations delivered within the target tidal volume (Vt), ventilation rate (Vr) or both (Table 3; Figure 2). The median risk ratio for improvement in compliance with target Vt across these studies was 2.32 (IQR: 1.83–2.78), while compliance with target Vr improved by a median of 1.84 (IQR: 1.76–1.90). The risk ratio for achieving compliance with both Vt and Vr combined was higher, with a median of 3.43 (IQR: 3.24–5.11). These findings suggest that while VFDs improve both aspects of ventilation, their effect may be more pronounced in improving tidal volume compliance than ventilation rate.

**Table 3.** Individual study results.

Author (Year)	Setting	Study country	Study type	VFD	Summary of findings	Participants		Vt in target			Vr in target			Vt and Vr in target		
						Control	VFD	Control	VFD	Risk ratio	Control	VFD	Risk ratio	Control	VFD	Risk ratio
Khoury et al. (2019)	Simulation	United States	Randomised crossover study	Custom	Improved ventilation performance (>70%) + minimised hyperventilation	40	40	Not reported								
Charlton et al. (2021)	Simulation	England	Before-and-after study	ZOLL AccuVent	Baseline ventilation quality frequently outside of recommendations, but VFD improved performance	106	106	22.65%	94.34%	4.17	51%	94.34%	1.85	9%	91%	10.11
Gould et al. (2020)	Simulation	United States	Before-and-after study	ZOLL AccuVent	Improved Vr and Vt accuracy	20	20	31% (11–51%)	79% (61–97%)	2.55	41% ±23% (SD)	71% ± 16% (SD)	1.73	16%	55%	3.44
Heo et al. (2020)	Simulation	South Korea	Randomised crossover study	Custom	Improved Vt and optimal Vr	13	13	18.46%	47.29%	2.56	50.20%	95.65%	1.91	Not reported		
Kim et al. (2020)	Simulation	South Korea	Randomised crossover study	Amflow	Appropriate Vt and Vr simultaneously in various scenarios	40	40	41.0%	85.4%	2.08	12.5%	99.2%	7.94	Not reported		
Scott et al. (2021)	Simulation	United States	Before-and-after study	ZOLL AccuVent	Tidal volume accuracy varied, though respiratory rate targets consistently met	52	52	42.3%	Not reported							
Lyngby et al. (2021)	Simulation	Denmark	Non-blinded randomised controlled trial	ZOLL AccuVent	Increased guideline compliance for Vr and Vt	32	32	53.4% (IQR 8.40–66.7%)	77.5 (IQR 64.9–83.8%)	1.45	66.7% (IQR 40.9–77.9%)	97.4% (IQR 97.1–100%)	1.46	22.1% (IQR 0–44.0%)	75.3% (IQR 66.2–82.9%)	3.41
Lee et al. (2023)	Clinical practice	South Korea	Randomised controlled study	ZOLL AccuVent	Improved ROSC + survival but no effect on neurological performance	58	63	Not reported								
Drennan et al. (2024)	Clinical practice	Canada	Before-and-after study	ZOLL AccuVent	Increased compliance with ventilation guidelines	191	221	21% (SD 16%)	28% (SD 17%)	1.33	29% (SD 19%)	53% (SD 38%)	1.83	7% (SD 10%)	19% (SD 17%)	2.71

Unlike these six studies, Khoury et al. (2019), Lee et al. (2023) and Scott et al. (2021) assessed different outcome measures, making direct comparisons with the compliance-focused studies difficult. However, Scott et al.’s findings help to explain trends seen in the compliance studies. They found that clinicians consistently met the target ventilation rate of 10 breaths per minute without audio or visual feedback, suggesting that respiratory rate is relatively easy to control. In contrast, tidal volume delivery varied significantly, with fewer than half of participants achieving the recommended 5–8 mL/kg, and 67.3% showing high variability between breaths. This aligns with the general trend across studies, where VFDs had a greater effect on tidal volume compliance than on ventilation rate.

Khoury et al. (2019) highlighted the issue of hyperventilation, demonstrating that VFD use significantly improved ventilation quality by reducing excessive ventilation. Compliance with recommended ventilation targets improved from 15% to 90% with a face mask and from 15% to 85% with an endotracheal tube, reinforcing the need for feedback to mitigate this common problem.

Lee et al. (2023) was the first study to investigate real-time audio ventilation feedback in a clinical setting rather than a simulation. Their findings suggest an association between VFD use and improved return of spontaneous circulation (ROSC) as well as short-term survival, marking an important step towards understanding the potential clinical benefits of these devices.

### VFDs in simulation

Six studies (Charlton et al., 2021; Drennan et al., 2024; Gould et al., 2020; Heo et al., 2020; Kim et al., 2020; Lyngby et al., 2021) utilised a VFD in a simulation setting. Simulation studies consistently demonstrated that manual ventilation without feedback often deviates from guidelines. Compliance rates for both ventilation rate and tidal volume range from as low as 9% to 66.7%, with hyperventilation being a common issue. The introduction of VFDs significantly improved adherence, with compliance increasing to between 47.2% and 99.2% across studies (see Table 3).

Among the five simulation studies that provided data on target compliance rates, the reported median risk ratios (RRs) for tidal volume were 2.55 (IQR: 1.77–3.4) and for ventilation rate, 1.85 (IQR: 1.60–4.93). The largest reported effect was an RR of 10–11 for combined compliance with ventilation rate and tidal volume in Charlton et al. (2021), while the smallest effect was 1.45 for tidal volume compliance in Lyngby et al. (2021). Notably, all simulation studies demonstrated a positive impact of VFD use. Beyond improved compliance, VFDs also reduced variability in ventilation delivery. Standard deviations (Gould et al., 2020) and interquartile ranges (Lyngby et al., 2021) were smaller in the VFD groups, indicating more consistent ventilation. Charlton et al. (2021) similarly showed that ventilation rate variability decreased, with ranges narrowing from 4–30 bpm without feedback to 6–17 bpm with feedback. Tidal volume variation was also reduced, from 201–1114 mL to 490–750 mL.

Scott et al. (2021) also assessed ventilation performance but did not provide results for pre- and post-feedback performance overall. Rather, their findings highlighted inconsistencies in ventilation delivery, with glove size and provider sex influencing tidal volume. While these findings support VFD effectiveness, the applicability of simulation studies to real-world OHCAs remains uncertain. Simulations lack the unpredictability of clinical settings, and the inability to blind participants, the crossover effect and the Hawthorn effect all introduce potential sources of systematic bias. Though efforts were taken by all study authors to minimise these effects, it is likely that the controlled environment of simulations overestimates VFD impact in practice. 

### VFDs in clinical trials

Two studies examined VFDs in real-world settings.

Lee et al. (2023) conducted a randomised controlled trial in Busan, South Korea, comparing standard defibrillators with those incorporating VFDs. Among 121 patients, they found improved ROSC and 30-hour survival rates (Lee et al., 2023). However, inconsistencies in reported odds ratios and exclusions prior to randomisation raise concerns about data integrity and study quality. We therefore deem this paper to be of low quality and cannot ascertain the effects on patient outcomes based on this study.

Drennan et al. (2024) performed a before-and-after study with 412 patients in Ontario, Canada. Paramedics initially used VFD-equipped defibrillators without real-time feedback, followed by a phase where feedback was provided. The study found significant improvements in ventilation compliance with VFD use, though the effect was smaller than in simulation studies. Rates of ROSC were similar between the intervention (n = 59, 27%) and control groups (n = 50, 29%). Exploratory multivariate logistic regression suggested that after adjusting for patient demographics, neither ventilation rate nor ventilation volume were associated with increased rates of ROSC. The authors suggested that additional implementation strategies are needed to optimise VFD effectiveness in clinical practice.

## Discussion

Our literature review provides a comprehensive overview of the current evidence surrounding the use of VFDs in OHCA scenarios. The findings suggest VFDs improve guideline compliance, potentially enhancing patient outcomes. In simulation settings, the use of VFDs increased the accuracy and precision of delivered ventilations, reducing instances of both hyperventilation and hypoventilation.

 The results of our review are consistent with previous studies indicating the challenges faced in maintaining appropriate ventilation rates and volumes during OHCA (Khoury et al., 2016; O’Neill and Deakin, 2007). Our findings build on this knowledge by demonstrating that real-time feedback can mitigate these challenges, aligning with the broader body of research that supports the use of feedback mechanisms to improve CPR quality (Gugelmin-Almeida et al., 2021; Lyngby et al., 2021).

In line with the broader literature on feedback, it appears that ventilation feedback is most effective when baseline performance is particularly poor. For example, Charlton et al. (2021) observed a substantial increase in guideline adherence from 9% to 91% with VFD use. This aligns with Ivers et al. (2012), who found that feedback interventions tend to have the greatest impact when initial performance is low, reinforcing the potential value of VFDs in clinical environments where ventilation quality is highly variable.

### Barriers to implementation

The results of the simulation studies appear to show that the use of VFDs is beneficial in improving guideline compliance; however, as highlighted by Drennan et al. (2024), there are several barriers to implementation that must be considered before their introduction into clinical practice.

### Training requirements

For the introduction of VFDs to be beneficial, clinicians must be adequately trained to effectively utilise this technology. This is emphasised by the study performed by Scott et al. (2021), in which participants were given ventilation feedback with no explanation or training. In a questionnaire completed at the end of the study, only 73.1% of participants were aware of the correct tidal volume that should be delivered – perhaps explaining why there did not appear to be a large increase in ventilation compliance.

The design of the VFD may impact the training requirements for its effective use. In the studies by Charlton et al. (2021), Gould et al. (2020) and Lyngby et al. (2021), the ZOLL AccuVent sensor was used, which has an interactive dashboard that clearly shows the correct tidal volume and respiratory rate for ventilation. This allowed for researchers to provide only a brief introduction to the device before the simulations. In Charlton et al. (2021), it was reported that 35% of participants found the brief training to be ‘sufficient’ and 65% found it to be ‘more than enough’. This highlights that the training requirements to utilise a VFD could be minimal and easily implemented at low cost.

### Information overload and the risk of harm

One concern raised was that the introduction of this technology may cause information overload, negatively affecting other aspects of the resuscitation.

In the studies performed by Charlton et al. (2021), Gould et al. (2020) and Lyngby et al. (2021), chest compression quality was measured and the introduction of the VFD had no negative effect on this, appearing to show that it did not lead to information overload. However, in these simulations the participants are only providing ventilations and compressions. This may be representative of the latter stages of an OHCA, but in the early stages there are often only a small number of clinicians who need to multitask, performing functions such as defibrillation and airway management, potentially making clinicians more susceptible to information overload.

It is unlikely that the introduction of VFDs poses a risk of harm. Overloaded clinicians may be more likely to ignore feedback rather than compromise resuscitation. This appears to be supported by a study performed by Wagner et al. (2022), which found that the use of feedback devices increased the participants’ self-reported subjective workload but did not negatively influence resuscitation quality.

### Lack of outcome data

Due to the limitations relating to Lee et al. (2023), there is no high-quality outcome data from the use of VFDs in clinical trials. This makes it difficult for organisations to justify their introduction into clinical practice.

Despite this, there is a strong theoretical argument that these devices may benefit patient outcomes. As well as showing an overall increase in guideline compliance, these studies also show that the introduction of VFDs led to improved accuracy and precision when delivering ventilations (Figure 2) and reduced extreme hypoventilation and hyperventilation, which is the most likely to have a negative effect.

### Limitations

Selection bias arose from including only studies published after 1 January 2018, potentially excluding relevant earlier research. Moreover, limiting the review to English studies may exclude valuable non-English data.

Study heterogeneity posed a major challenge. Variations in study design, sample sizes and settings (such as simulation versus clinical settings) made direct comparisons difficult and limited the generalisability of findings across different contexts.

To focus on high-quality interventional evidence, we excluded observational and descriptive studies. While this approach aimed to ensure robust conclusions, it may have missed important insights from real-world clinical practice.

Our review was constrained by a limited number of studies meeting the inclusion criteria. With only nine studies included, predominantly simulation-based with two clinical studies, the small sample size restricts the strength of our conclusions and emphasises the need for additional research, particularly in real-world clinical settings.

Publication bias is a concern in our review, as studies with positive results are more likely to be published than those with negative or inconclusive findings. This bias can influence the overall interpretation of the effectiveness of VFDs. For example, despite the recent completion of the VANZ2 study on VFD use in UK clinical practice, no published evidence was located, illustrating this potential bias.

Most of the studies included in our review were conducted in simulated environments. While valuable, these findings may not fully replicate the complexities and unpredictability of real-world OHCA scenarios, limiting their direct applicability to clinical practice.

Inconsistencies were noted in the reporting of outcomes across the reviewed clinical study. These discrepancies raise concerns about data reliability and underscore the importance of standardised reporting in future research. Standardisation would facilitate more accurate comparisons and meta-analyses across studies in this field.

## Conclusion

This literature review aimed to establish if the evidence base supports the introduction of a real-time ventilation feedback device into clinical practice. Nine studies were selected for inclusion. Seven of these studies were deemed to be high quality and demonstrated good reliability and validity, while the others had several flaws and have been treated as low-quality evidence.

This review suggests that clinicians frequently provide ventilations that do not comply with guidelines. The introduction of VFDs resulted in significantly higher rates of ventilation guideline compliance in simulated cardiac arrests, reducing both hypoventilation and hyperventilation. In real world settings, the introduction of VFDs was associated with higher rates of compliance, though the effect was smaller than in simulated studies and further research is needed before rolling out VFDs in clinical practice.

## Author contributions

CB conceived and designed the study, undertook data searches and screening, analysed the data and drafted the first version of the manuscript. AC supervised the design and undertaking of the initial study and reviewed and edited the manuscript. CW analysed the data and reviewed and edited the manuscript. CB acts as the guarantor for this article.

## Conflict of interest

CW is an associate editor for the BPJ.

## Ethics

Not required.

## Funding

None.

## Statement of generative AI in scientific writing

The authors did not use a generative artificial intelligence (AI) tool or service to assist with preparation or editing of this work.
